# Supplementary material for: How cells wrap around virus-like particles using extracellular filamentous protein structures
Source: bioRxiv. 2023 Jan 30:2023.01.30.526272. Preprint. [Version 1] doi: 10.1101/2023.01.30.526272 (PMC9915516; doi:10.1101/2023.01.30.526272)
Supplement: Supplement 1 [file NIHPP2023.01.30.526272v1-supplement-1.pdf]

bioRxiv preprint doi: https://doi.org/10.1101/2023.01.30.526272; this version posted January 30, 2023. The copyright holder for this preprint (which was not certified by peer review) is the author/funder, who has granted bioRxiv a license to display the preprint in perpetuity. It is made available under aCC-BY 4.0 International license.

I. SUPPLEMENTARY FIGURES

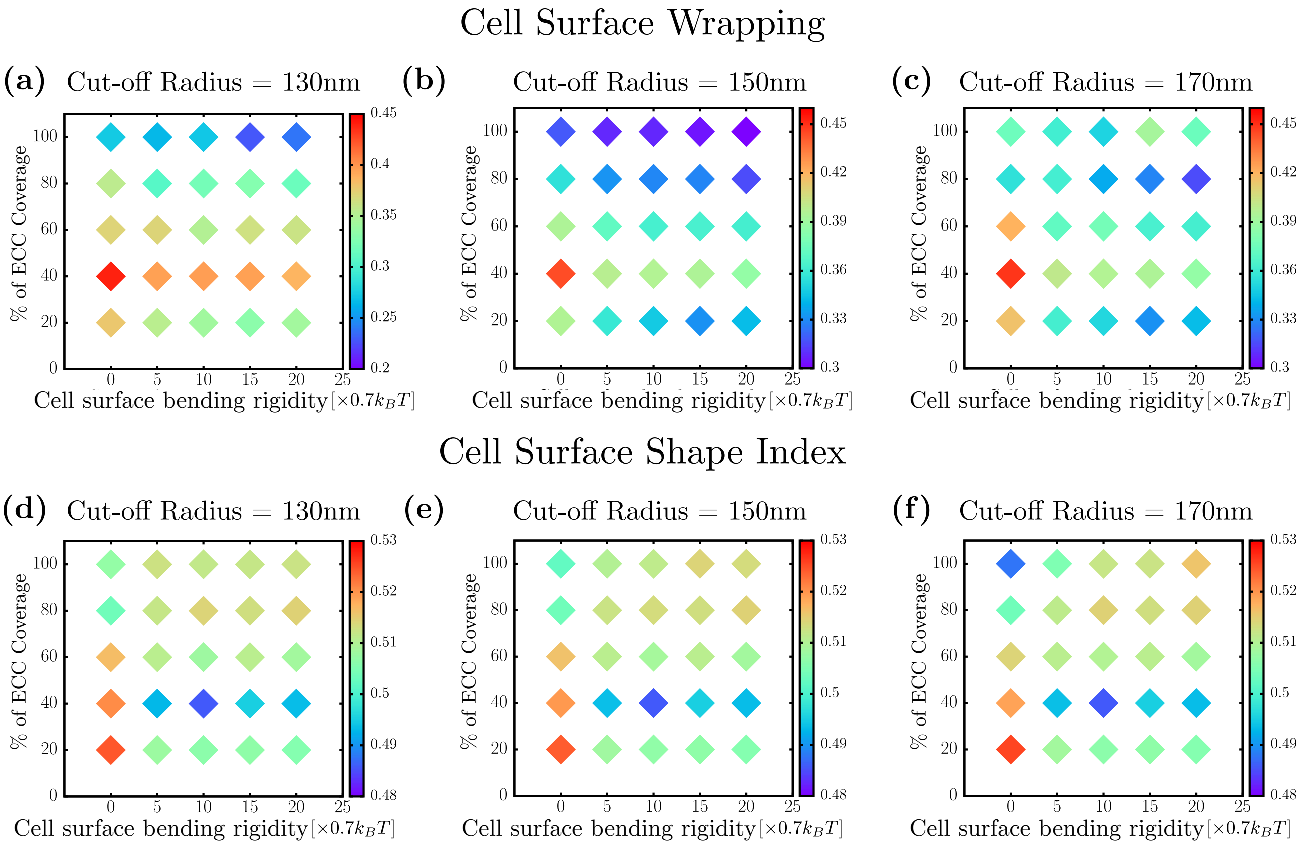

FIG. S1. Cell surface wrapping and shape index is robust with the varying cylinder's radius cut-off

bioRxiv preprint doi: https://doi.org/10.1101/2023.01.30.526272; this version posted January 30, 2023. The copyright holder for this preprint (which was not certified by peer review) is the author/funder, who has granted bioRxiv a license to display the preprint in perpetuity. It is made available under aCC-BY 4.0 International license.

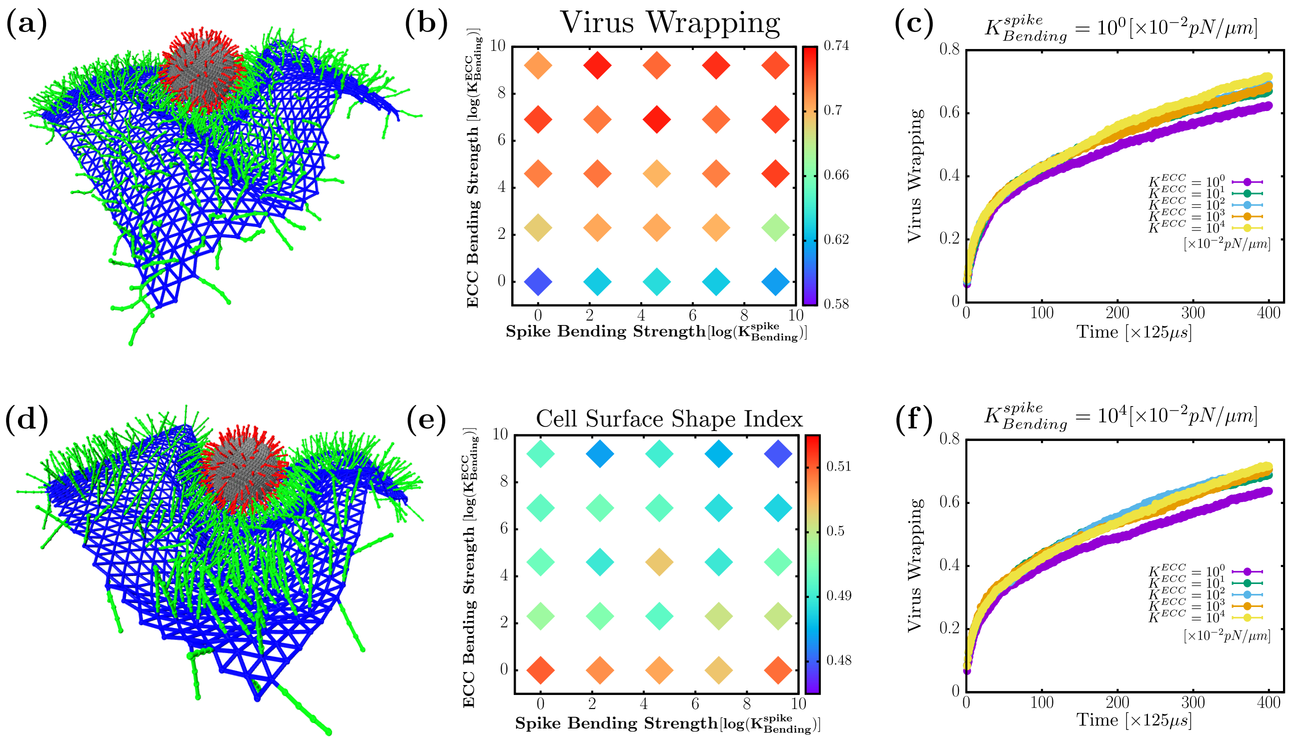

FIG. S2. *Varying ECC and spike bending rigidities does not lead to cell surface crumples* (a) Simulation snapshot for $K^{spike}_{Bending} = 10^0$ and $K^{ECC}_{Bending} = 10^0$ show folded state (b) log-log plot: Heat map of virus wrapping showing at low ECC bending strength irrespctive of spike bending strength gives low wrapping compared to high ECC bending strength (c) and (f) Virus wrapping as a function of time for $K^{spike}_{Bending} = 10^0$ and $K^{spike}_{Bending} = 10^4$ for various values of $K^{ECC}_{Bending}$ (d) Simulation snapshot for $K^{spike}_{Bending} = 10^4$ and $K^{ECC}_{Bending} = 10^4$ showing sharply folded cell surface (e) log-log plot: Heat map of cell surface shape index have a higher value at low bending strength of spike indicating soft folds formation, but at high ECC bending strength has lower shape index points to sharp fold formation. Biologically relevant time units can be obtained by multiplying 125μs that follow from the time units defined previously.

bioRxiv preprint doi: https://doi.org/10.1101/2023.01.30.526272; this version posted January 30, 2023. The copyright holder for this preprint (which was not certified by peer review) is the author/funder, who has granted bioRxiv a license to display the preprint in perpetuity. It is made available under aCC-BY 4.0 International license.

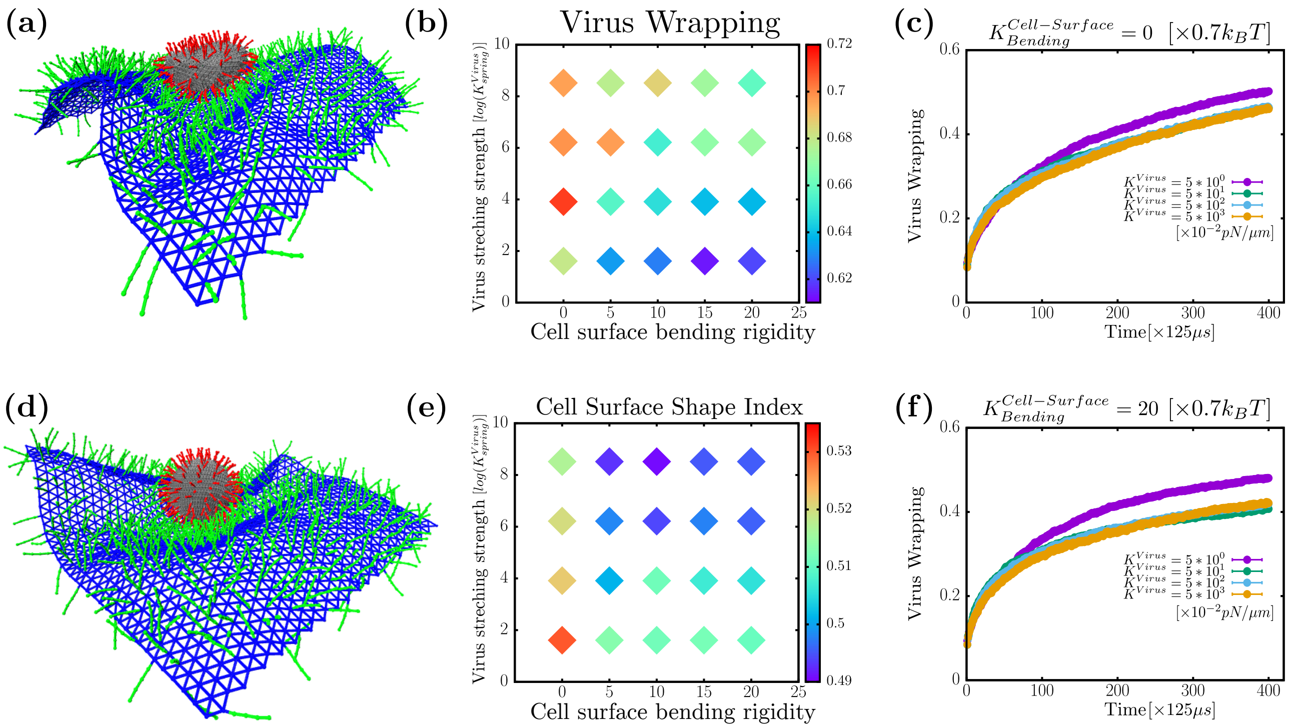

FIG. S3. *Varying virus stretching rigidity* (a) Simulation snapshot for $K^{Virus}_{Spring} = 5 * 10^0$ (b) log-log plot: Heat map of virus wrapping as a function of virus stretching strength and cell surface bending rigidity. Since the virus is deformable, virus wrapping here is defined as the ratio of occupied spikes divided by the total number of virus spikes. (c) and (f) Virus wrapping as a function of time for $K^{Cell-Surface}_{Bending} = 0$ and $K^{Cell-Surface}_{Bending} = 20$ for various values of K^{Virus}_{Spring} (d) Simulation snapshot for $K^{Virus}_{Spring} = 5 * 10^4$ (e) log-log plot: Heat map of cell surface shape index as a function of virus stretching strength and cell surface bending rigidity. Biologically relevant time units can be obtained by multiplying $125\mu s$ that follow from the time units defined previously.
